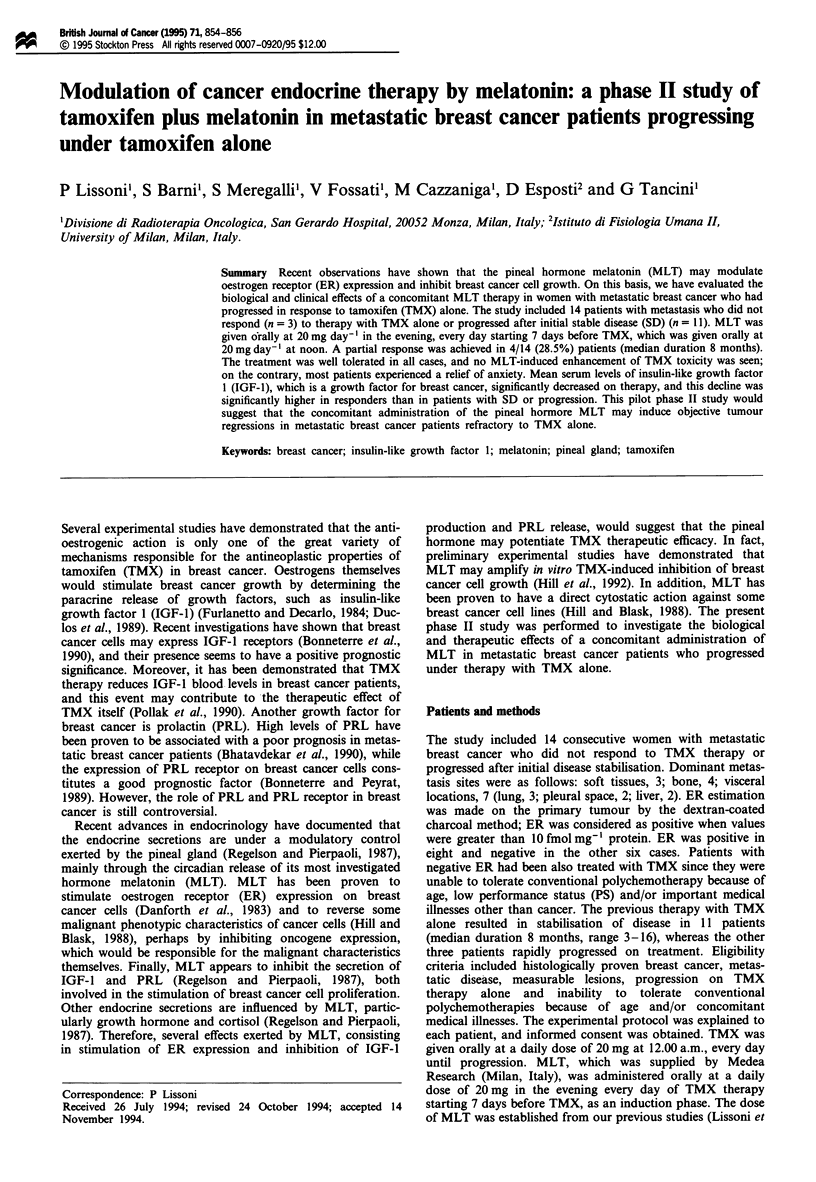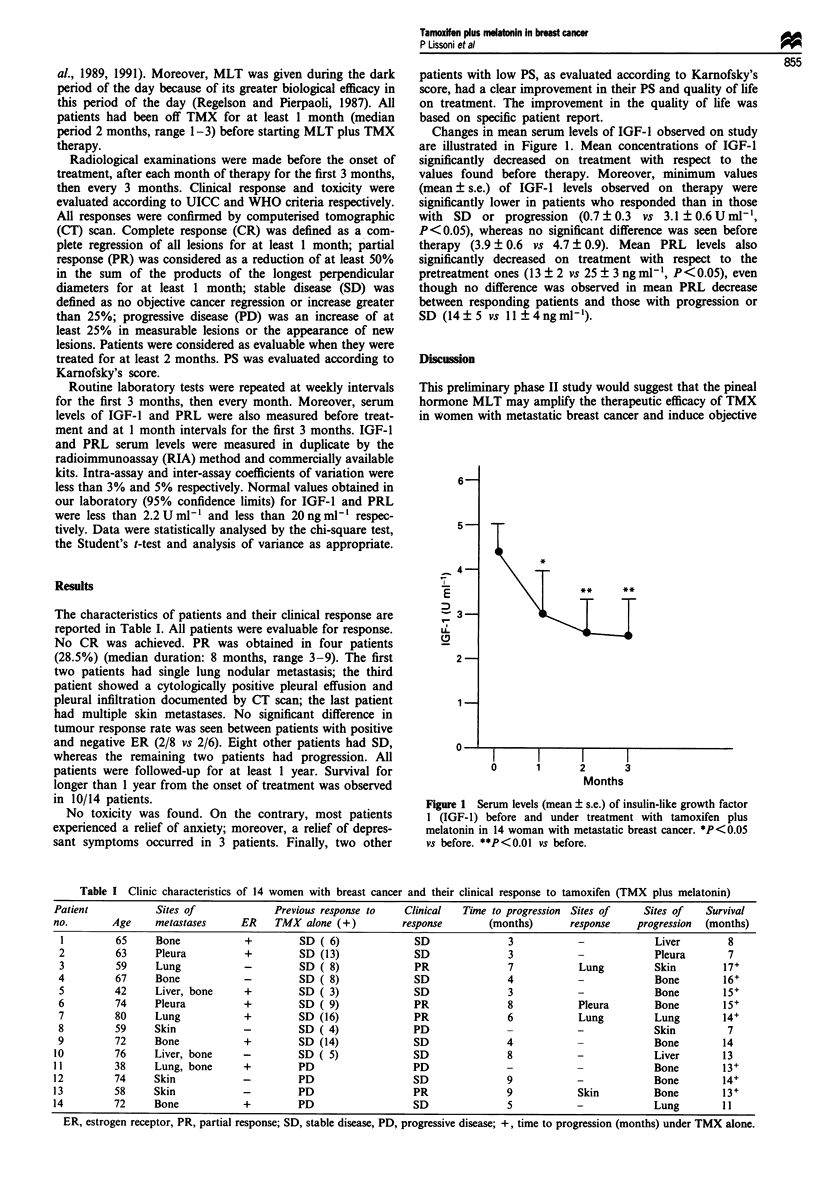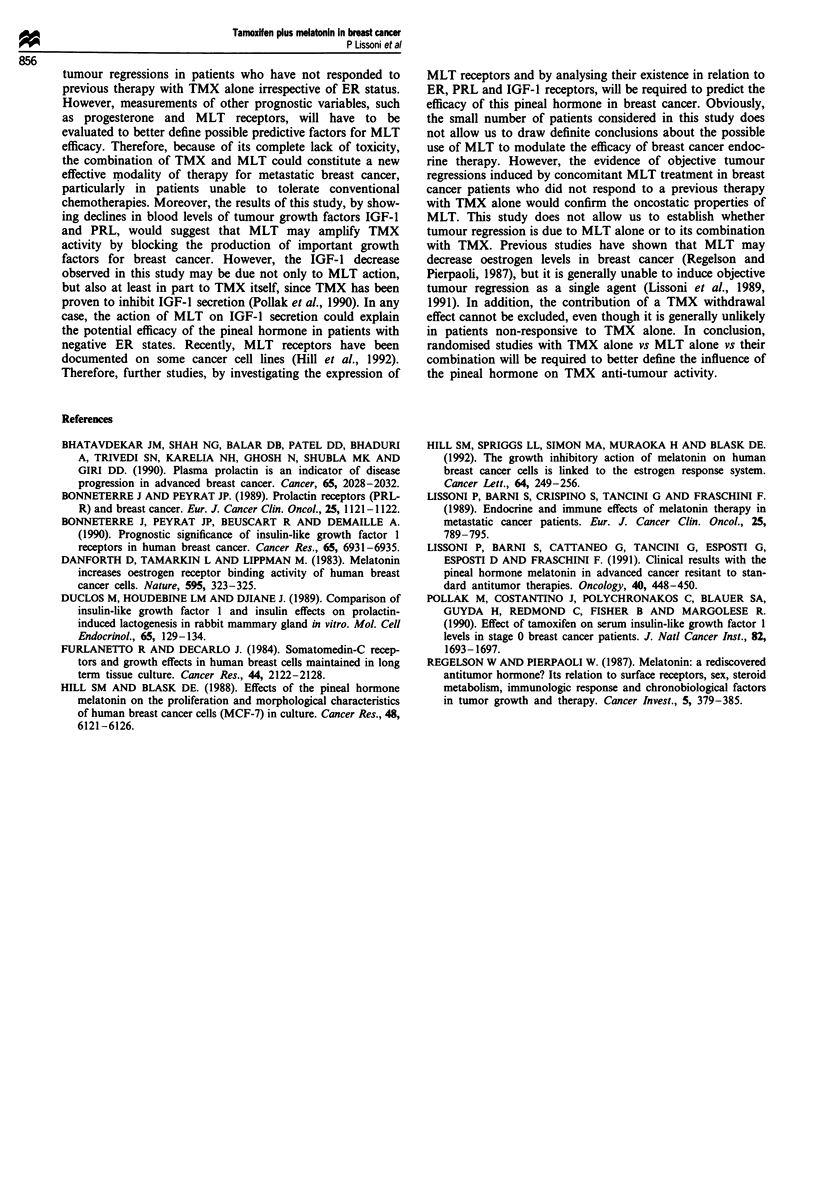# Modulation of cancer endocrine therapy by melatonin: a phase II study of tamoxifen plus melatonin in metastatic breast cancer patients progressing under tamoxifen alone.

**DOI:** 10.1038/bjc.1995.164

**Published:** 1995-04

**Authors:** P. Lissoni, S. Barni, S. Meregalli, V. Fossati, M. Cazzaniga, D. Esposti, G. Tancini

**Affiliations:** Divisione di Radioterapia Oncologica, San Gerardo Hospital, Monza, Milan, Italy.

## Abstract

Recent observations have shown that the pineal hormone melatonin (MLT) may modulate oestrogen receptor (ER) expression and inhibit breast cancer cell growth. On this basis, we have evaluated the biological and clinical effects of a concomitant MLT therapy in women with metastatic breast cancer who had progressed in response to tamoxifen (TMX) alone. The study included 14 patients with metastasis who did not respond (n = 3) to therapy with TMX alone or progressed after initial stable disease (SD) (n = 11). MLT was given orally at 20 mg day-1 in the evening, every day starting 7 days before TMX, which was given orally at 20 mg day-1 at noon. A partial response was achieved in 4/14 (28.5%) patients (median duration 8 months). The treatment was well tolerated in all cases, and no MLT-induced enhancement of TMX toxicity was seen; on the contrary, most patients experienced a relief of anxiety. Mean serum levels of insulin-like growth factor 1 (IGF-1), which is a growth factor for breast cancer, significantly decreased on therapy, and this decline was significantly higher in responders than in patients with SD or progression. This pilot phase II study would suggest that the concomitant administration of the pineal hormone MLT may induce objective tumour regressions in metastatic breast cancer patients refractory to TMX alone.


British Journal of Cancer (1995) 71, 854-856
© 1995 Stockton Press All rights reserved 0007-0920/95 $12.00

### Modulation of cancer endocrine therapy by melatonin: a phase II study of tamoxifen plus melatonin in metastatic breast cancer patients progressing under tamoxifen alone

P Lissoni[1], S Barni[1], S Meregalli[1], V Fossati[1], M Cazzaniga[1], D Esposti[2] and G Tancini[1]

[1]*Divisione di Radioterapia Oncologica, San Gerardo Hospital, 20052 Monza, Milan, Italy;* [2]*Istituto di Fisiologia Umana II, University of Milan, Milan, Italy.*

**Summary** Recent observations have shown that the pineal hormone melatonin (MLT) may modulate oestrogen receptor (ER) expression and inhibit breast cancer cell growth. On this basis, we have evaluated the biological and clinical effects of a concomitant MLT therapy in women with metastatic breast cancer who had progressed in response to tamoxifen (TMX) alone. The study included 14 patients with metastasis who did not respond ($n = 3$) to therapy with TMX alone or progressed after initial stable disease (SD) ($n = 11$). MLT was given orally at 20 mg day$^{-1}$ in the evening, every day starting 7 days before TMX, which was given orally at 20 mg day$^{-1}$ at noon. A partial response was achieved in 4/14 (28.5%) patients (median duration 8 months). The treatment was well tolerated in all cases, and no MLT-induced enhancement of TMX toxicity was seen; on the contrary, most patients experienced a relief of anxiety. Mean serum levels of insulin-like growth factor 1 (IGF-1), which is a growth factor for breast cancer, significantly decreased on therapy, and this decline was significantly higher in responders than in patients with SD or progression. This pilot phase II study would suggest that the concomitant administration of the pineal hormore MLT may induce objective tumour regressions in metastatic breast cancer patients refractory to TMX alone.

**Keywords:** breast cancer; insulin-like growth factor 1; melatonin; pineal gland; tamoxifen

Several experimental studies have demonstrated that the antioestrogenic action is only one of the great variety of mechanisms responsible for the antineoplastic properties of tamoxifen (TMX) in breast cancer. Oestrogens themselves would stimulate breast cancer growth by determining the paracrine release of growth factors, such as insulin-like growth factor 1 (IGF-1) (Furlanetto and Decarlo, 1984; Duclos *et al.*, 1989). Recent investigations have shown that breast cancer cells may express IGF-1 receptors (Bonneterre *et al.*, 1990), and their presence seems to have a positive prognostic significance. Moreover, it has been demonstrated that TMX therapy reduces IGF-1 blood levels in breast cancer patients, and this event may contribute to the therapeutic effect of TMX itself (Pollak *et al.*, 1990). Another growth factor for breast cancer is prolactin (PRL). High levels of PRL have been proven to be associated with a poor prognosis in metastatic breast cancer patients (Bhatavdekar *et al.*, 1990), while the expression of PRL receptor on breast cancer cells constitutes a good prognostic factor (Bonneterre and Peyrat, 1989). However, the role of PRL and PRL receptor in breast cancer is still controversial.

Recent advances in endocrinology have documented that the endocrine secretions are under a modulatory control exerted by the pineal gland (Regelson and Pierpaoli, 1987), mainly through the circadian release of its most investigated hormone melatonin (MLT). MLT has been proven to stimulate oestrogen receptor (ER) expression on breast cancer cells (Danforth *et al.*, 1983) and to reverse some malignant phenotypic characteristics of cancer cells (Hill and Blask, 1988), perhaps by inhibiting oncogene expression, which would be responsible for the malignant characteristics themselves. Finally, MLT appears to inhibit the secretion of IGF-1 and PRL (Regelson and Pierpaoli, 1987), both involved in the stimulation of breast cancer cell proliferation. Other endocrine secretions are influenced by MLT, particularly growth hormone and cortisol (Regelson and Pierpaoli, 1987). Therefore, several effects exerted by MLT, consisting in stimulation of ER expression and inhibition of IGF-1 production and PRL release, would suggest that the pineal hormone may potentiate TMX therapeutic efficacy. In fact, preliminary experimental studies have demonstrated that MLT may amplify *in vitro* TMX-induced inhibition of breast cancer cell growth (Hill *et al.*, 1992). In addition, MLT has been proven to have a direct cytostatic action against some breast cancer cell lines (Hill and Blask, 1988). The present phase II study was performed to investigate the biological and therapeutic effects of a concomitant administration of MLT in metastatic breast cancer patients who progressed under therapy with TMX alone.

### Patients and methods

The study included 14 consecutive women with metastatic breast cancer who did not respond to TMX therapy or progressed after initial disease stabilisation. Dominant metastasis sites were as follows: soft tissues, 3; bone, 4; visceral locations, 7 (lung, 3; pleural space, 2; liver, 2). ER estimation was made on the primary tumour by the dextran-coated charcoal method; ER was considered as positive when values were greater than 10 fmol mg$^{-1}$ protein. ER was positive in eight and negative in the other six cases. Patients with negative ER had been also treated with TMX since they were unable to tolerate conventional polychemotherapy because of age, low performance status (PS) and/or important medical illnesses other than cancer. The previous therapy with TMX alone resulted in stabilisation of disease in 11 patients (median duration 8 months, range 3–16), whereas the other three patients rapidly progressed on treatment. Eligibility criteria included histologically proven breast cancer, metastatic disease, measurable lesions, progression on TMX therapy alone and inability to tolerate conventional polychemotherapies because of age and/or concomitant medical illnesses. The experimental protocol was explained to each patient, and informed consent was obtained. TMX was given orally at a daily dose of 20 mg at 12.00 a.m., every day until progression. MLT, which was supplied by Medea Research (Milan, Italy), was administered orally at a daily dose of 20 mg in the evening every day of TMX therapy starting 7 days before TMX, as an induction phase. The dose of MLT was established from our previous studies (Lissoni *et*

Correspondence: P Lissoni
Received 26 July 1994; revised 24 October 1994; accepted 14 November 1994.

al., 1989, 1991). Moreover, MLT was given during the dark period of the day because of its greater biological efficacy in this period of the day (Regelson and Pierpaoli, 1987). All patients had been off TMX for at least 1 month (median period 2 months, range 1-3) before starting MLT plus TMX therapy.

Radiological examinations were made before the onset of treatment, after each month of therapy for the first 3 months, then every 3 months. Clinical response and toxicity were evaluated according to UICC and WHO criteria respectively. All responses were confirmed by computerised tomographic (CT) scan. Complete response (CR) was defined as a complete regression of all lesions for at least 1 month; partial response (PR) was considered as a reduction of at least 50% in the sum of the products of the longest perpendicular diameters for at least 1 month; stable disease (SD) was defined as no objective cancer regression or increase greater than 25%; progressive disease (PD) was an increase of at least 25% in measurable lesions or the appearance of new lesions. Patients were considered as evaluable when they were treated for at least 2 months. PS was evaluated according to Karnofsky's score.

Routine laboratory tests were repeated at weekly intervals for the first 3 months, then every month. Moreover, serum levels of IGF-1 and PRL were also measured before treatment and at 1 month intervals for the first 3 months. IGF-1 and PRL serum levels were measured in duplicate by the radioimmunoassay (RIA) method and commercially available kits. Intra-assay and inter-assay coefficients of variation were less than 3% and 5% respectively. Normal values obtained in our laboratory (95% confidence limits) for IGF-1 and PRL were less than 2.2 U $ml^{-1}$ and less than 20 ng $ml^{-1}$ respectively. Data were statistically analysed by the chi-square test, the Student's $t$-test and analysis of variance as appropriate.

### Results

The characteristics of patients and their clinical response are reported in Table I. All patients were evaluable for response. No CR was achieved. PR was obtained in four patients (28.5%) (median duration: 8 months, range 3-9). The first two patients had single lung nodular metastasis; the third patient showed a cytologically positive pleural effusion and pleural infiltration documented by CT scan; the last patient had multiple skin metastases. No significant difference in tumour response rate was seen between patients with positive and negative ER (2/8 vs 2/6). Eight other patients had SD, whereas the remaining two patients had progression. All patients were followed-up for at least 1 year. Survival for longer than 1 year from the onset of treatment was observed in 10/14 patients.

No toxicity was found. On the contrary, most patients experienced a relief of anxiety; moreover, a relief of depressant symptoms occurred in 3 patients. Finally, two other patients with low PS, as evaluated according to Karnofsky's score, had a clear improvement in their PS and quality of life on treatment. The improvement in the quality of life was based on specific patient report.

Changes in mean serum levels of IGF-1 observed on study are illustrated in Figure 1. Mean concentrations of IGF-1 significantly decreased on treatment with respect to the values found before therapy. Moreover, minimum values (mean ± s.e.) of IGF-1 levels observed on therapy were significantly lower in patients who responded than in those with SD or progression (0.7 ± 0.3 vs 3.1 ± 0.6 U $ml^{-1}$, $P < 0.05$), whereas no significant difference was seen before therapy (3.9 ± 0.6 vs 4.7 ± 0.9). Mean PRL levels also significantly decreased on treatment with respect to the pretreatment ones (13 ± 2 vs 25 ± 3 ng $ml^{-1}$, $P < 0.05$), even though no difference was observed in mean PRL decrease between responding patients and those with progression or SD (14 ± 5 vs 11 ± 4 ng $ml^{-1}$).

### Discussion

This preliminary phase II study would suggest that the pineal hormone MLT may amplify the therapeutic efficacy of TMX in women with metastatic breast cancer and induce objective

**Figure 1** Serum levels (mean ± s.e.) of insulin-like growth factor 1 (IGF-1) before and under treatment with tamoxifen plus melatonin in 14 woman with metastatic breast cancer. $^{*}P < 0.05$ vs before. $^{**}P < 0.01$ vs before.

**Table I** Clinic characteristics of 14 women with breast cancer and their clinical response to tamoxifen (TMX plus melatonin)

| *Patient no.* | *Age* | *Sites of metastases* | *ER* | *Previous response to TMX alone* (+) | *Clinical response* | *Time to progression* (months) | *Sites of response* | *Sites of progression* | *Survival* (months) |
|---|---|---|---|---|---|---|---|---|---|
| 1 | 65 | Bone | + | SD ( 6) | SD | 3 | – | Liver | 8 |
| 2 | 63 | Pleura | + | SD (13) | SD | 3 | – | Pleura | 7 |
| 3 | 59 | Lung | – | SD ( 8) | PR | 7 | Lung | Skin | 17⁺ |
| 4 | 67 | Bone | – | SD ( 8) | SD | 4 | – | Bone | 16⁺ |
| 5 | 42 | Liver, bone | + | SD ( 3) | SD | 3 | – | Bone | 15⁺ |
| 6 | 74 | Pleura | + | SD ( 9) | PR | 8 | Pleura | Bone | 15⁺ |
| 7 | 80 | Lung | + | SD (16) | PR | 6 | Lung | Lung | 14⁺ |
| 8 | 59 | Skin | – | SD ( 4) | PD | – | – | Skin | 7 |
| 9 | 72 | Bone | + | SD (14) | SD | 4 | – | Bone | 14 |
| 10 | 76 | Liver, bone | – | SD ( 5) | SD | 8 | – | Liver | 13 |
| 11 | 38 | Lung, bone | + | PD | PD | – | – | Bone | 13⁺ |
| 12 | 74 | Skin | – | PD | SD | 9 | – | Bone | 14⁺ |
| 13 | 58 | Skin | – | PD | PR | 9 | Skin | Bone | 13⁺ |
| 14 | 72 | Bone | + | PD | SD | 5 | – | Lung | 11 |

ER, estrogen receptor, PR, partial response; SD, stable disease, PD, progressive disease; +, time to progression (months) under TMX alone.

tumour regressions in patients who have not responded to previous therapy with TMX alone irrespective of ER status. However, measurements of other prognostic variables, such as progesterone and MLT receptors, will have to be evaluated to better define possible predictive factors for MLT efficacy. Therefore, because of its complete lack of toxicity, the combination of TMX and MLT could constitute a new effective modality of therapy for metastatic breast cancer, particularly in patients unable to tolerate conventional chemotherapies. Moreover, the results of this study, by showing declines in blood levels of tumour growth factors IGF-1 and PRL, would suggest that MLT may amplify TMX activity by blocking the production of important growth factors for breast cancer. However, the IGF-1 decrease observed in this study may be due not only to MLT action, but also at least in part to TMX itself, since TMX has been proven to inhibit IGF-1 secretion (Pollak *et al*., 1990). In any case, the action of MLT on IGF-1 secretion could explain the potential efficacy of the pineal hormone in patients with negative ER states. Recently, MLT receptors have been documented on some cancer cell lines (Hill *et al*., 1992). Therefore, further studies, by investigating the expression of MLT receptors and by analysing their existence in relation to ER, PRL and IGF-1 receptors, will be required to predict the efficacy of this pineal hormone in breast cancer. Obviously, the small number of patients considered in this study does not allow us to draw definite conclusions about the possible use of MLT to modulate the efficacy of breast cancer endocrine therapy. However, the evidence of objective tumour regressions induced by concomitant MLT treatment in breast cancer patients who did not respond to a previous therapy with TMX alone would confirm the oncostatic properties of MLT. This study does not allow us to establish whether tumour regression is due to MLT alone or to its combination with TMX. Previous studies have shown that MLT may decrease oestrogen levels in breast cancer (Regelson and Pierpaoli, 1987), but it is generally unable to induce objective tumour regression as a single agent (Lissoni *et al*., 1989, 1991). In addition, the contribution of a TMX withdrawal effect cannot be excluded, even though it is generally unlikely in patients non-responsive to TMX alone. In conclusion, randomised studies with TMX alone *vs* MLT alone *vs* their combination will be required to better define the influence of the pineal hormone on TMX anti-tumour activity.

### References

BHATAVDEKAR JM, SHAH NG, BALAR DB, PATEL DD, BHADURI A, TRIVEDI SN, KARELIA NH, GHOSH N, SHUBLA MK AND GIRI DD. (1990). Plasma prolactin is an indicator of disease progression in advanced breast cancer. *Cancer*, **65**, 2028–2032.

BONNETERRE J AND PEYRAT JP. (1989). Prolactin receptors (PRL-R) and breast cancer. *Eur. J. Cancer Clin. Oncol*., **25**, 1121–1122.

BONNETERRE J, PEYRAT JP, BEUSCART R AND DEMAILLE A. (1990). Prognostic significance of insulin-like growth factor 1 receptors in human breast cancer. *Cancer Res*., **65**, 6931–6935.

DANFORTH D, TAMARKIN L AND LIPPMAN M. (1983). Melatonin increases oestrogen receptor binding activity of human breast cancer cells. *Nature*, **595**, 323–325.

DUCLOS M, HOUDEBINE LM AND DJIANE J. (1989). Comparison of insulin-like growth factor 1 and insulin effects on prolactin-induced lactogenesis in rabbit mammary gland *in vitro*. *Mol. Cell Endocrinol*., **65**, 129–134.

FURLANETTO R AND DECARLO J. (1984). Somatomedin-C receptors and growth effects in human breast cells maintained in long term tissue culture. *Cancer Res*., **44**, 2122–2128.

HILL SM AND BLASK DE. (1988). Effects of the pineal hormone melatonin on the proliferation and morphological characteristics of human breast cancer cells (MCF-7) in culture. *Cancer Res*., **48**, 6121–6126.

HILL SM, SPRIGGS LL, SIMON MA, MURAOKA H AND BLASK DE. (1992). The growth inhibitory action of melatonin on human breast cancer cells is linked to the estrogen response system. *Cancer Lett*., **64**, 249–256.

LISSONI P, BARNI S, CRISPINO S, TANCINI G AND FRASCHINI F. (1989). Endocrine and immune effects of melatonin therapy in metastatic cancer patients. *Eur. J. Cancer Clin. Oncol*., **25**, 789–795.

LISSONI P, BARNI S, CATTANEO G, TANCINI G, ESPOSTI G, ESPOSTI D AND FRASCHINI F. (1991). Clinical results with the pineal hormone melatonin in advanced cancer resitant to standard antitumor therapies. *Oncology*, **40**, 448–450.

POLLAK M, COSTANTINO J, POLYCHRONAKOS C, BLAUER SA, GUYDA H, REDMOND C, FISHER B AND MARGOLESE R. (1990). Effect of tamoxifen on serum insulin-like growth factor 1 levels in stage 0 breast cancer patients. *J. Natl Cancer Inst*., **82**, 1693–1697.

REGELSON W AND PIERPAOLI W. (1987). Melatonin: a rediscovered antitumor hormone? Its relation to surface receptors, sex, steroid metabolism, immunologic response and chronobiological factors in tumor growth and therapy. *Cancer Invest*., **5**, 379–385.